# CD43, but not CD41 or GPI-80, marks first definitive haematopoietic stem cells in the human embryo

**DOI:** 10.1242/dev.205108

**Published:** 2025-11-20

**Authors:** Andrejs Ivanovs, Stanislav Rybtsov, Nneka Nnadi, Adrien Fayon, Sabrina Gordon-Keylock, Daria Paruzina, Richard A. Anderson, Manuela Tavian, Alexander Medvinsky

**Affiliations:** ^1^ Ontogeny of Haematopoietic Stem Cells Group, Institute for Stem Cell Research, Centre for Regenerative Medicine, Institute for Regeneration and Repair, University of Edinburgh, Edinburgh EH16 4UU, UK; ^2^ Department of Morphology, Institute of Anatomy and Anthropology, Riga Stradiņš University, Riga LV-1010, Latvia; ^3^ Université de Strasbourg, Inserm, IRFAC/UMR-S1113, FMTS, Strasbourg 67200, France; ^4^ Centre for Reproductive Health, Institute for Regeneration and Repair, University of Edinburgh, Edinburgh EH16 4UU, UK; ^5^ Université de Strasbourg, INSERM UMR-S1109, FMTS, Strasbourg 67200, France

**Keywords:** AGM region, Human embryo, Haematopoietic stem cell, Dorsal aorta

## Abstract

During mammalian embryonic development, haematopoietic stem cells (HSCs) first emerge in the aorta-gonad-mesonephros (AGM) region. Human definitive HSCs emerge in low numbers and reside within the VE-CAD^+^CD45^+^ population consisting of 500-1000 cells. Accurate identification of the first HSCs emerging within this population is important for understanding their biology and underlying developmental mechanisms. Here, we characterised the expression of potential markers labelling HSCs during their emergence in the AGM at Carnegie stages (CS) 14-17. We found that the first definitive HSCs are marked by CD43, but not CD41, similar to the early haematopoietic progenitor cells differentiating in culture from human embryonic stem cells. We show that, during development in the AGM region and in the beginning of liver colonisation, HSCs remain GPI-80 negative, in contrast to liver HSCs at the later midtrimester foetal development. Together with our previous observations, this study provides firm evidence that HSCs colonise human embryonic liver at CS17-18.

## Teaser

**Summary:** Analysis of human HSCs reveals that the first definitive HSCs emerging in the AGM region during CS14-17 exhibit a distinct marker profile compared with midtrimester HSCs, and that HSCs colonise human embryonic liver at CS17-18.

## INTRODUCTION

The first human definitive (transplantable) haematopoietic stem cells (HSCs) emerge in the embryonic aorta-gonad-mesonephros (AGM) region during CS14-17. These rare cells capable of long-term repopulation of adult recipients reside within the CD34^+^VE-CAD (CDH5)^+^CD45 (PTPRC)^+^c-KIT^+^THY1^+^endoglin (ENG)^+^RUNX1^+^CD38^−/lo^CD45RA^−^ population localised to the ventral domain of the dorsal aorta (Ivanovs et al., 2014). As in the mouse, human HSCs initially emerge in low numbers: one or two HSCs per AGM region (Kumaravelu et al., 2002; Ivanovs et al., 2011). While cell sorting combined with *in vivo* long-term repopulation assays enables considerable enrichment of human umbilical cord blood HSCs (Majeti et al., 2007; Notta et al., 2011; Knapp et al., 2018; Anjos-Afonso et al., 2022), currently purification of only around one AGM-derived HSC per 500-1000 CD34^+^VE-CAD^+^CD45^+^ cells, depending on developmental stage, can be achieved (Ivanovs et al., 2014). This large population also contains committed haematopoietic progenitors (CFU-Cs) and likely immature HSC precursors (pre-HSCs) undetectable by direct long-term transplantations (Ivanovs et al., 2014; Rybtsov et al., 2014).

In this study, we investigated whether additional surface proteins, CD41 (ITGA2B), CD43 (SPN) and GPI-80 (VNN2), known to mark HSCs at some stages of mammalian development, are expressed by human AGM region-derived HSCs. In the mouse, CD41 marks early embryonic haematopoietic progenitors and immature embryonic day (E)9.5 HSC precursors (pro-HSCs), which by E10.5 upregulate CD43 (pre-HSC I) and subsequently CD45 (pre-HSC II) (Ferkowicz et al., 2003; Mikkola et al., 2003; Taoudi et al., 2008; Rybtsov et al., 2011, 2014; Zhou et al., 2016). In human embryonic stem cells, haematopoietic specification of haematogenic endothelium is marked by upregulation of CD43 and only later by CD41 (Vodyanik et al., 2006). Using long-term transplantations into immunocompromised adult NOD.Cg-Prkdc^scid^Il2rg^tm1^Wjl/Sz (NSG) mice, we show that the first human AGM-derived definitive HSCs are marked by CD43, but not by CD41. Additionally, although GPI-80 is expressed by human midtrimester foetal liver HSCs (Prashad et al., 2015; Vanuytsel et al., 2022), it does not mark the first transplantable HSCs from human AGM. We conclude that, in addition to the previously reported phenotype (CD34^+^VE-CAD^+^CD45^+^c-KIT^+^THY1^+^ENG^+^RUNX1^+^CD38^−/lo^CD45RA^−^), the first definitive human HSCs are characterised by the expression of CD43^+^ and the absence of CD41 and GPI-80 expression. Additionally, taken together with previous observations, this study provides final firm evidence that colonisation of the embryonic liver by definitive HSCs occurs at Carnegie stages (CS) 17-18 (Ivanovs et al., 2011, 2020; Easterbrook et al., 2019).

## RESULTS

### Kinetics of CD43, CD41, GPI-80 and c-KIT expression in hematopoietic/endothelial populations of the human AGM region

**Fig. 1.**
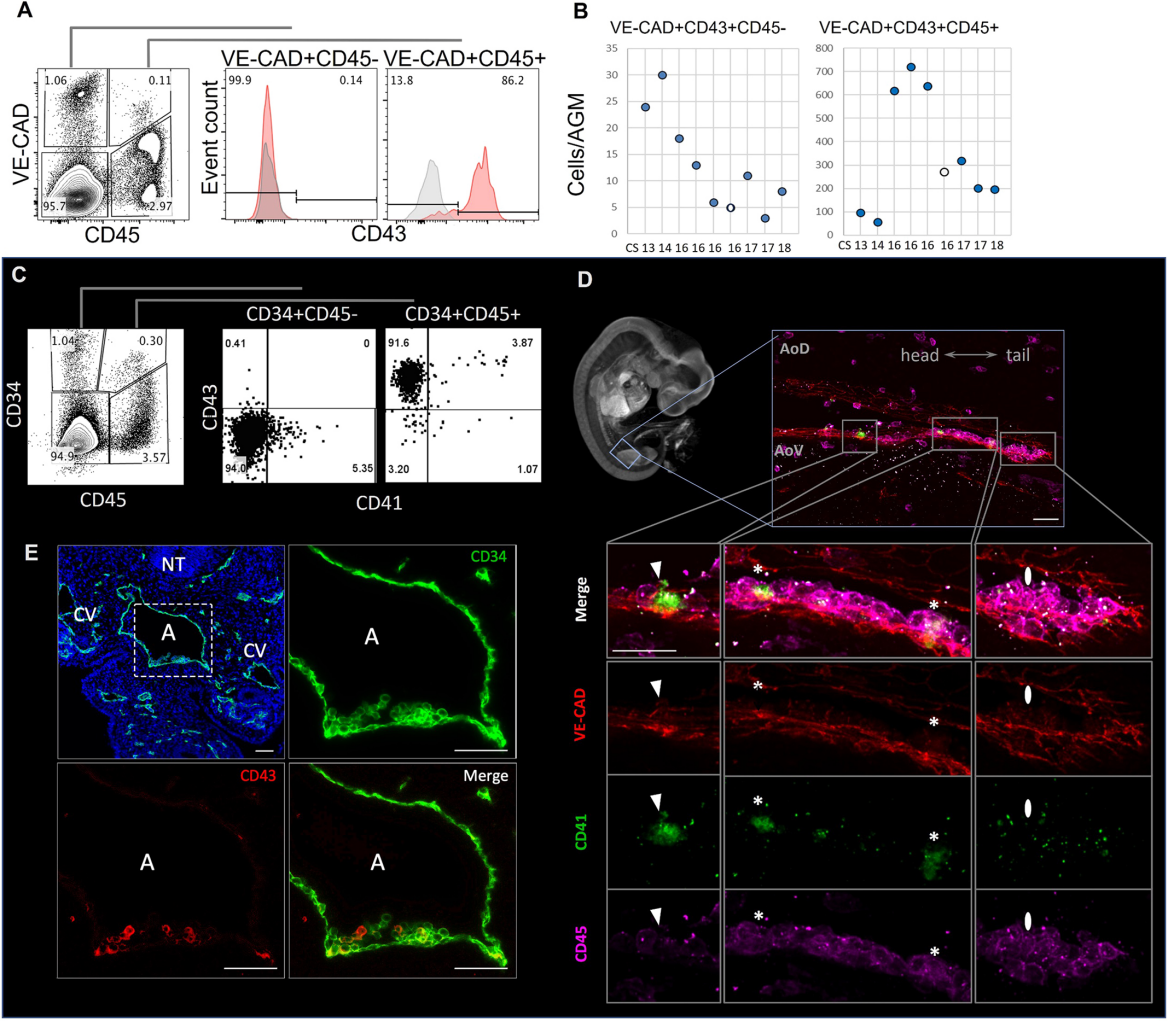
**CD43 and CD41 expression in haemato-endothelial populations of the human AGM region.** (A) CD43 expression in the VE-CAD^+^CD45^−^ and VE-CAD^+^CD45^+^ populations (representative example of a CS16 human embryo). (B) Temporal kinetics of CD43 expression in VE-CAD^+^CD45^−^ and VE-CAD^+^CD45^+^ populations, depending upon the stage of development (bottom of the graphs). Note, the plots do not imply age differences between embryos represented by the same CS. (C) CD43 expression in the CD34^+^CD45^−^ and CD34^+^CD45^+^ populations (representative example of a CS16 human embryo). (D) CD41 expression in IAHCs on the ventral wall of the dorsal aorta (whole-mount staining of a CS14 human embryo). AoD, dorsal domain of the dorsal aorta; AoV, ventral domain of the dorsal aorta. The left-most cluster contains VE-CAD^+^CD41^+^CD45^−^ cells (shown by arrowheads); the large middle cluster contains VE-CAD^+^CD41^+^CD45^+^ (shown by asterisks); the right-most cluster contains VE-CAD^+^CD41^−^CD45^+^ cells (shown by ovals). (E) CD43 expression in IAHCs on the ventral wall of the dorsal aorta. Transverse sections of CS14 embryo AGM region immunostained for CD34 and CD43 (*n*=4 independent embryos). A, aorta; CV, cardinal vein; NT, neural tube. Boxed area is shown at higher magnification in the other three panels. Scale bars: 50 μm.

Flow cytometry analysis showed that during CS13-18, the majority of endothelial (VE-CAD^+^CD45^−^) cells in the AGM region are CD43 negative (Fig. 1A; Table S1). At most, 25 cells (<1.2%) of the entire endothelial VE-CAD^+^CD45^−^ population expressed CD43, supposedly marking the initiation of the endothelial-to-haematopoietic transition (EHT) (Fig. 1B). By contrast, the VE-CAD^+^CD45^+^ haematopoietic stem and progenitor cell (HSPC) population (500-1000 cells per AGM region) enriched for haematopoietic progenitors and immature HSCs contained 47-95% of CD43^+^ cells depending on the developmental stage (Fig. 1A,B; Table S1). The kinetics of CD43 expression differed between the VE-CAD^+^CD45^−^ and the VE-CAD^+^CD45^+^ populations over the CS13-18 period. A small fraction of CD43^+^ cells in the endothelial VE-CAD^+^CD45^−^ population at CS13-14, became virtually undetectable at CS16-17, whereas in the HSPC VE-CAD^+^CD45^+^ population the initially small CD43^+^ fraction peaked at CS16 and dropped dramatically by CS17-18 (Fig. 1B). Gradual decline of the CD43^+^ cell numbers in the endothelial population likely reflects progressive upregulation of CD45 that shifts them to the HSPC pool. Notably, CS15-16 is the time of the most robust detectability of HSCs in the AGM region (Ivanovs et al., 2011).

The analysis of CD41 expression showed that within the AGM-derived CD34^+^CD45^+^ population (also expressing VE-CAD), CD41 marks only up to ∼4% of cells, which potentially makes it a good HSC enrichment marker (Fig. 1C). We further examined the expression of CD43 and CD41 by immunofluorescence in human embryonic sections. Both markers were detected on cells within intra-aortic haematopoietic cell clusters (IAHCs) with heterogeneous expression patterns consistent with our flow cytometry analysis (Fig. 1C-E; Figs S1, S2, Table S1). The observed heterogeneity in CD43, CD41 and CD45 expression may reflect the step-wise EHT progression or/and parallel independent production of HSCs and transient progenitors from distinct types of haematogenic endothelial cells (Heck et al., 2020; Zhu et al., 2020; Randolph et al., 2025).

**Fig. 2.**
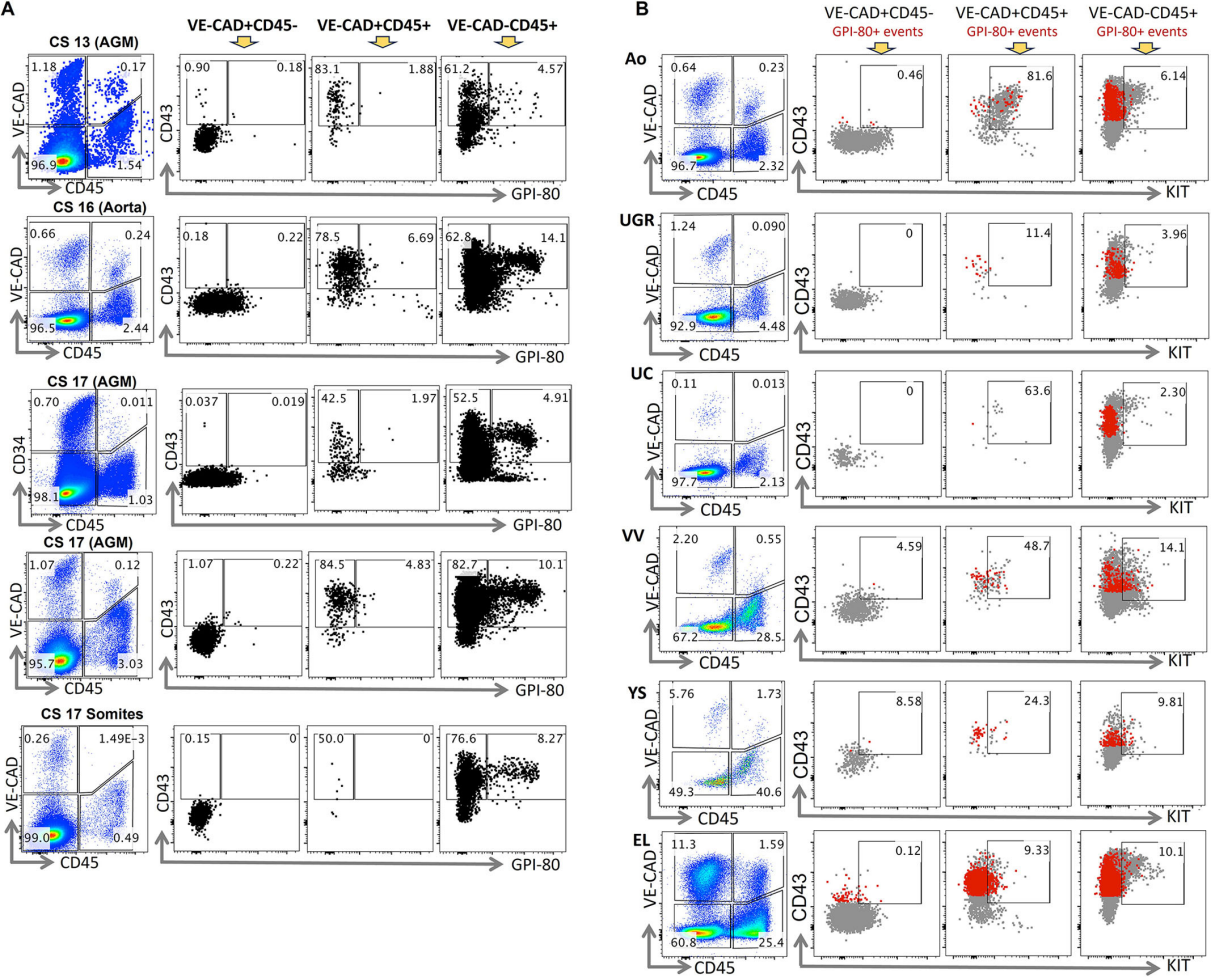
**GPI-80 and c-KIT expression in haemato-endothelial populations of the human AGM region.** (A) GPI-80 and CD43 expression in the VE-CAD^+^CD45^−^, VE-CAD^+^CD45^+^ and VE-CAD^−^CD45^+^ cell populations in haematopoietic (AGM region and dorsal aorta) and non-haematopoietic (somites) tissues (representative examples of CS13-17 human embryos). (B) c-KIT and CD43 expression in the VE-CAD^+^CD45^−^, VE-CAD^+^CD45^+^ and VE-CAD^−^CD45^+^ cell population in the dorsal aorta (Ao), uro-genital ridges (UGR), umbilical cord (UC), vitelline vessels (VV), yolk sac (YS) and embryonic liver (EL) (representative example of a CS16 human embryo). GPI-80^+^ events are depicted in red.

We then characterised the expression of GPI-80, a marker which enabled fourfold enrichment of HSCs within the midtrimester human foetal liver CD34^+^CD38^−/lo^THY1^+^ population (Prashad et al., 2015). At CS13, prior to emergence of definitive HSCs in the AGM region, GPI-80 was not detected in endothelial VE-CAD^+^CD45^−^ cells and was virtually absent in the VE-CAD^+^CD45^+^ HSPC population. Only at CS16-17, approximately 2-7% of VE-CAD^+^CD45^+^ HSPC cells showed weak GPI-80 expression (Fig. 2A). Meanwhile, more mature VE-CAD^−^CD45^+^ blood cells already at CS13 exhibited broader and stronger GPI-80^+^ expression (4-14% cells by CS16), consistent with expression of this marker on myeloid cells (Takeda et al., 2003). Most cells in both VE-CAD^+^CD45^+^ and VE-CAD^−^CD45^+^ populations that were GPI-80^+^ also expressed CD43. Somites were virtually devoid of VE-CAD^+^CD45^+^ cells, consistent with the lack of both EHT and HSC generation in this site, but contained mature VE-CAD^−^CD45^+^ cells co-expressing CD43 and GPI-80 (Fig. 2A).

We previously reported that the stem cell factor (SCF) receptor c-KIT, a well-established marker of human umbilical cord blood HSCs (Kawashima et al., 1996; Maillard et al., 2020), labels the entire VE-CAD^+^CD45^+^ population in the AGM region (Ivanovs et al., 2014). Here, we found that in some embryos a subset of aorta VE-CAD^+^CD45^+^ cells lack c-KIT expression, which might represent non-HSC progenitors generated during EHT and/or reflect variabilities in temporal differentiation dynamics between embryos (Fig. 2B). In extra-embryonic umbilical cord (UC) and vitelline vessels (VVs), c-KIT expression in VE-CAD^+^CD45^+^ populations was considerable, 64% and 49%, respectively (Fig. 2B), but not total. In contrast, in secondary haematopoietic sites, c-KIT^+^ fractions within the VE-CAD^+^CD45^+^ populations were either almost absent (uro-genital ridges) or significantly reduced (yolk sac and embryonic liver). At all developmental stages, the majority of VE-CAD^+^CD45^+^c-KIT^+^ cells in the aorta was marked by CD43, but more rarely by GPI-80. In extra-embryonic vessels (mainly VVs) and the yolk sac the VE-CAD^+^CD45^+^c-KIT+ population was smaller than in the aorta and GPI-80 expression was lower. Although relatively abundant in the embryonic liver, this population showed weaker GPI-80 expression than in the aorta (Fig. 2B).

Expression of distinct CD41^+^, CD43^+^ and GPI-80^+^ fractions within the AGM VE-CAD^+^CD45^+^ population poses the question of which of these antigens mark HSCs. To address this question, we first transcriptionally profiled these populations.

### Transcriptional signature of AGM region in HSPCs is GPI-80 deficient

**Fig. 3.**
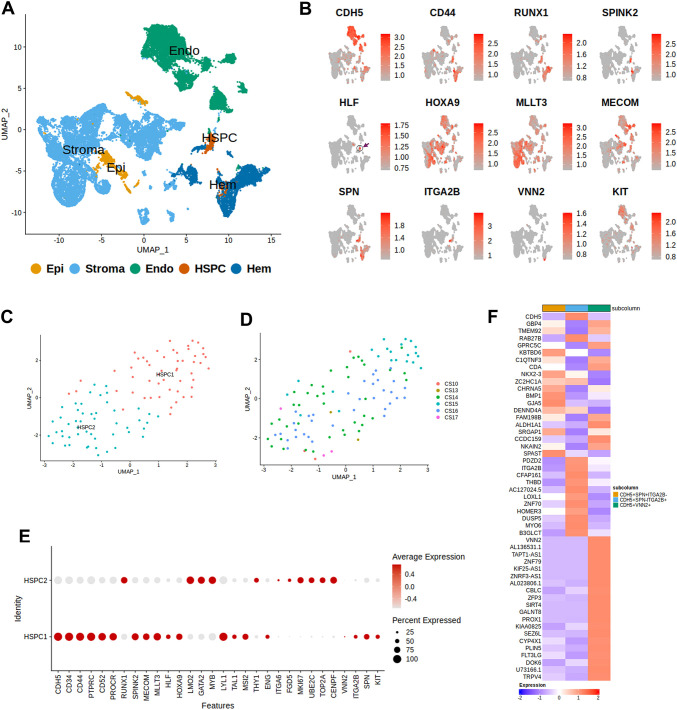
**Transcriptional signature of AGM HSPCs.** (A) UMAP visualisation of the CS10-17 datasets (Zeng et al., 2019; Crosse et al., 2020, 2023; Calvanese et al., 2022) listed in Table S2. Five major cell categories are annotated based on the gene expression listed in Table S3. (B). Feature plots of genes enriched in developing HSCs and the markers under question: *SPN* (CD43), *ITGA2B* (CD41), *VNN2* (GPI-80). A small cell subset expressing the proposed HSC signature markers is outlined by a circle and arrow in the *HLF* plot. (C) HSPC populations represented by HSPC1 and HSPC2 sub-populations (re-clustering from Fig. 3A). See also Table S4. (D) HSPC populations as in C across the developmental time points. (E) Dot plot of differential expression of selected genes across HSPC1 and HSPC2 sub-populations. (F) Heatmap showing differential gene expression between VE-CAD^+^ populations expressing *SPN*, *ITAGB* and *VNN2* (see also Table S5).

We have re-analysed previously published single-cell RNA-sequencing data for CS14-17 dorsal aorta to align with our immunophenotypic analysis (Table S2) (Zeng et al., 2019; Crosse et al., 2020, 2023; Calvanese et al., 2022). Unsupervised clustering revealed five major cell populations: endothelial, HSPCs, mature haematopoietic, stroma cells and epithelial cells, as defined by key markers (Fig. 3A; Table S3). The HSPC cluster was enriched for expression of *RUNX1*, *CD34*, *HLF*, *MECOM*, *HOXA9*, *MLLT3*, *SPINK2*, previously proposed to constitute the developing HSC signature (Calvanese et al., 2022). As expected, this molecular signature was restricted to a small subset of cells best defined by focused *HLF* expression (Fig. 3B).

Consistent with our previous report, HSPCs were subdivided into two sub-populations, HSPC1 and HSPC2, of which HSPC1 displayed a more quiescent phenotype as assessed by lower expression of the proliferative markers *MKI67*, *UBE2C*, *TOP2A* and *CENPF* compared to HSPC2 (Fig. 3C,D; Table S4) (Crosse et al., 2023). All genes constituting the proposed developing HSC signature, except *RUNX1*, were expressed considerably higher in HSPC1, including *HOXA9*, *HLF* and *MLLT3*, which are important for HSC function (Fig. 3E) (Lawrence et al., 2005; Komorowska et al., 2017; Calvanese et al., 2019, 2022; Lynch et al., 2024). Additionally, PROCR (also known as EPCR), a marker for developing and adult mouse and human HSCs, was also expressed more highly in HSPC1 (Schulte et al., 2015; Zhou et al., 2016; Fares et al., 2017; Subramaniam et al., 2019; Rabe et al., 2020; Anjos-Afonso et al., 2022). In contrast, HSPC2 showed higher expression of *RUNX1*, *LMO2*, *GATA2*, *MYB* and the surface marker *THY1* (Fig. 3E; Table S4). Preferential expression of the proposed HSC signature genes suggests that HSCs may reside within the more quiescent HSPC1 population.

Further analysis showed that *SPN* (CD43), *ITGA2B* (CD41) and *KIT* are mapped mainly to the HSPC1 fraction, thus co-expressing with the proposed HSC signature genes (Fig. 3E). *VNN2* (GPI-80) expression was detected only in a tiny proportion of cells, but also more prominently in HSPC1 than in HSPC2. Transcriptional profiling did not reveal prevalent expression of the signature genes in neither VE-CAD^+^CD43^+^CD41^−^ nor VE-CAD^+^CD43^−^CD41^+^ nor VE-CAD^+^GPI-80^+^ cell populations. However, some notable differences between these populations were observed (Fig. 3F; Table S5). The VE-CAD^+^CD43^+^ population showed relatively high expression of the arterial marker *GJA5*, the transcription factor NKX2-3 and the TGFβ family protein BMP1. The other two populations (VE-CAD^+^CD41^+^ and VE-CAD^+^GPI-80^+^) also showed specific differences in gene expression, including expression of the zinc finger transcription factors *ZNF79* and *PROX1*, as well as secreted *FLT3LG* involved in proliferation of early haematopoietic cells. Although these differences suggest functional heterogeneity among the subsets, transcriptomic analysis alone is insufficient to identify the markers that reliably enrich for HSCs. Functional validation is required to determine the haematopoietic potential of these populations.

### Long-term repopulation shows that the first definitive HSCs are CD43^+^CD41^−^GPI-80^−^

**Fig. 4.**
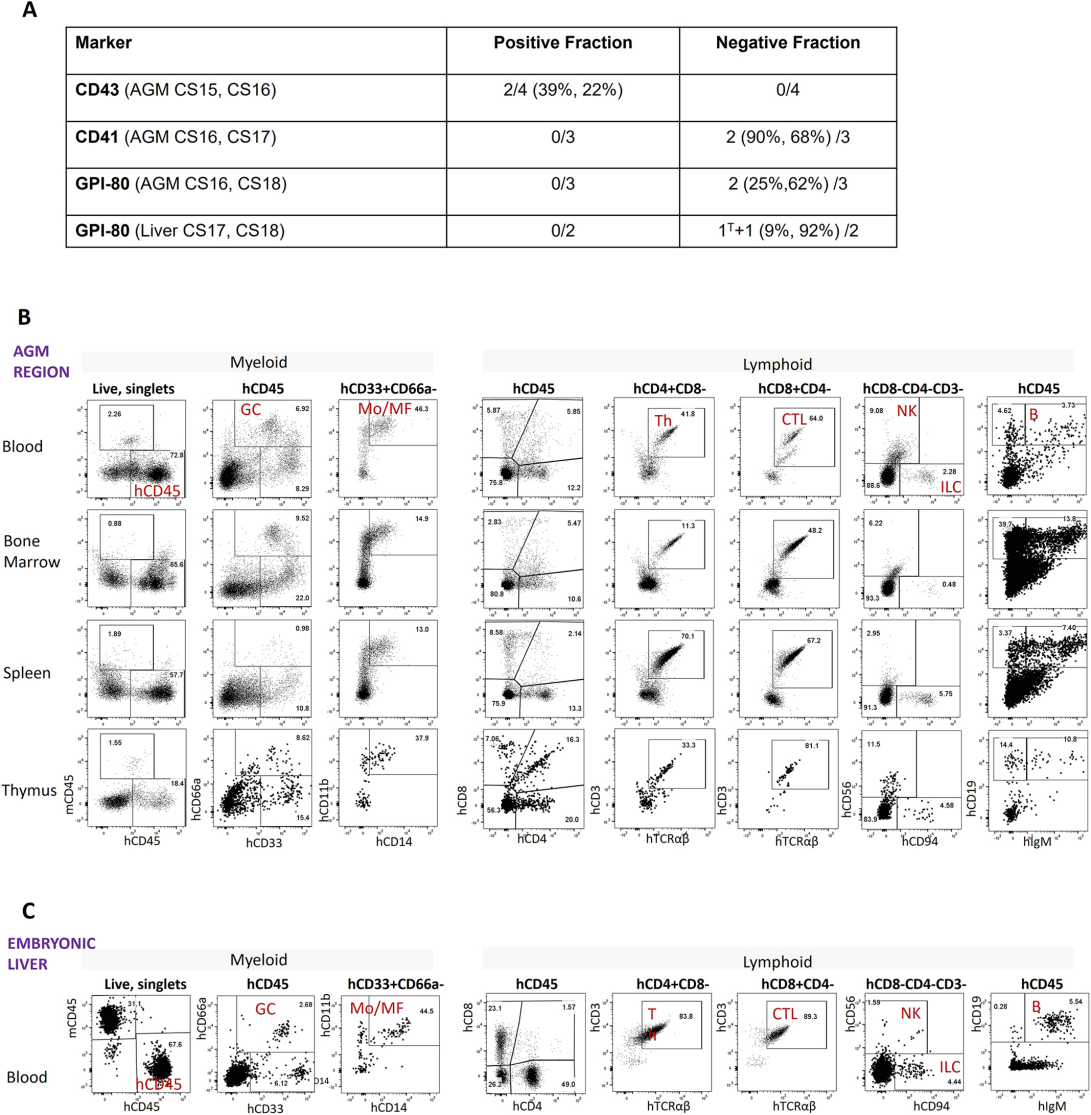
**Multilineage analysis of recipient NSG mice reconstituted by purified populations of human AGM region and liver cells.** (A) Long-term repopulation summary by purified cell populations: repopulated mice (percentage human chimerism)/total transplanted. Each AGM was transplanted into one or two mice per cell fraction. Each liver was transplanted into one recipient per cell fraction. All repopulated recipients showed multi-lineage differentiation, except one mouse (1^T^) transplanted with GPI-80-negative CS17 liver fraction, which showed uni-lineage T-cell repopulation. (B) Repopulation by GPI-80-negative cells purified from the CS17 AGM region (peripheral blood, bone marrow, spleen, thymus. (C) Repopulation by GPI-80-negative cells purified from CS18 liver (peripheral blood). B, B-lymphocytes; CTL, cytotoxic T-lymphocytes; GC, granulocytes; ILC, potentially innate lymphoid; MF, macrophages; Mo, monocytes; NK, NK cells; Th, T-helpers.

The first definitive HSCs within the human AGM region reside in the VE-CAD^+^CD45^+^ population (Ivanovs et al., 2014). To functionally determine expression of CD43, CD41 and GPI-80 in these cells, single-cell suspensions were prepared in separate experiments from eight CS15-18 human AGM regions, as well as one CS17 and one CS18 human embryonic livers. Cell populations were purified using magnetic beads and transplanted into sub-lethally irradiated adult NSG recipients as described previously (Ivanovs et al., 2011). The recipients were monitored for 4-6 months and considered to be engrafted if at least 0.1% of peripheral blood CD45^+^ cells were of human origin belonging to both myeloid and lymphoid lineages (results summarised in Fig. 4). Only experiments in which long-term engraftment was observed are discussed here.

In two experiments using purified AGM-derived CD43^+^ and CD43^−^ cell fractions, long-term donor-derived repopulation was observed exclusively in the recipients transplanted with the CD43^+^ fraction (Fig. 4A). Since approximately 80-90% of VE-CAD^+^CD45^+^ cells are CD43^+^, this marker provides modest enrichment for HSCs. In two other independent experiments based on CD41 expression, long-term human-derived repopulation was achieved only with CD41^−^ cells (which constitute ∼95% of VE-CAD^+^CD45^+^ cells), also providing a modest HSC enrichment.

To evaluate GPI-80 expression in HSCs, we performed four experiments using AGM and embryonic liver purified cells (Fig. 4A). In two experiments with the AGM region (CS16 and CS18), long-term multilineage repopulation was observed only in recipients of the GPI-80-negative fraction (Fig. 4B). Similarly, in two experiments with embryonic livers, only GPI-80^−^ cells showed repopulation, of which CS17 liver gave uni-lineage T-cell engraftment, whereas CS18 liver showed multilineage repopulation (Fig. 4C). In addition to standard lymphoid and myeloid lineages assayed in previous reports (Ivanovs et al., 2011, 2014), our current analysis revealed that HSCs from both the AGM region and the embryonic liver produced CD3^+^CD56 (NCAM)^+^ NK cells and phenotypic CD3^+^CD56^−^CD94 (KLRD1)^+^ innate lymphoid cells 6 months post-transplantation. Thus, early HSCs both in the AGM region and early embryonic liver lack GPI-80 expression, although later at midtrimester liver HSCs become GPI-80 positive (Prashad et al., 2015; Vanuytsel et al., 2022).

## DISCUSSION

During embryogenesis, gradual HSC lineage development within the AGM region culminates in the emergence of definitive (engraftable) HSCs. Insights into HSPC development can be inferred to some extent from computational single-cell transcriptomics analyses (Baron et al., 2018; Zeng et al., 2019; Crosse et al., 2020; Vink et al., 2020; Azzoni et al., 2021; Calvanese et al., 2022; Fadlullah et al., 2022; Hadland et al., 2022; Goh et al., 2023). However, despite its value, this approach cannot replace functional long-term repopulation assays, which remain the gold standard for identification of HSCs. In the mouse, at least three sequential classes of HSC embryonic precursors (pro/pre-HSCs) have been identified using AGM cultures that recapitulate HSC development (Taoudi et al., 2008; Rybtsov et al., 2011, 2014; Zhou et al., 2016; Ganuza et al., 2017; Hadland et al., 2022). The lack of an equivalent culture system for the human AGM region limits analysis to functional assaying of definitive HSCs only (Easterbrook et al., 2019). Along with IAHCs, HSCs emerge and disappear during the CS14-17/18 time-window covered in the present study (Tavian et al., 1999; Ivanovs et al., 2011).

In both the mouse and human AGM regions, HSCs reside within the VE-CAD^+^CD45^+^ population, which emerges during EHT (Taoudi et al., 2005; Ivanovs et al., 2014). In this study, we focused on additional markers: CD41 and CD43, which are expressed in early mouse pre- and definitive HSCs, and GPI-80 expressed in midtrimester human foetal liver HSCs (Rybtsov et al., 2014; Prashad et al., 2015; Vanuytsel et al., 2022). Both flow cytometry and immunofluorescence analyses showed heterogeneous co-expression of CD43, CD41 and GPI-80 during early haematopoietic specification in the AGM region. To obtain deeper insight into expression of these markers, we re-analysed publicly available single-cell transcriptomics datasets, and identified two HSPC clusters within the VE-CAD^+^CD45^+^ population: less proliferative (HSPC1) and more proliferative (HSPC2), consistent with our previous report (Crosse et al., 2023). Expression of recently proposed HSC molecular signature transcription factors, HOXA9, HLF and MLLT3 (Calvanese et al., 2022) and of PROCR, a surface marker expressed in HSCs, was more prominent in the more quiescent HSPC1 sub-population, indicating that HSCs may reside there. Some other factors playing a role in HSC biology, such as RUNX1, TEL and GATA2, are expressed predominantly in the HSPC2 sub-population. Transcriptomics analysis of VE-CAD^+^CD43^+^, VE-CAD^+^CD41^+^ and VE-CAD^+^GPI-80^+^ cell populations did not reveal clear association of the HSC signature markers with CD43, CD41 or GPI-80 expression.

Subsequent long-term transplantation experiments showed that definitive HSCs in the AGM region are CD43 positive but CD41 negative. Of note, CD43, but not CD41, marks the first haematopoietic cells emerging during human embryonic stem cell differentiation (Vodyanik et al., 2006). In our study, we observed a minor fraction of CD43^+^ cells emerging from the endothelium at CS13-14 progressively acquiring CD45 expression. The growing VE-CAD^+^CD45^+^ population peaks at CS16 and declines by CS17-18 likely due to the relocation to the liver. This timing aligns with IAHC and HSC developmental dynamics (detectability) in the AGM region (Tavian et al., 1999; Ivanovs et al., 2011). Our analysis indicates that CD43 inclusion in the staining panel can give 9-53% HSC enrichment depending on the CS (Fig. S2). Given the presence of one HSC per AGM region, this corresponds to an HSC enrichment between ∼1/56 and 1/638 VE-CAD^+^CD45^+^CD43^+^ cells depending on CS (Table S1). The inclusion of CD41 and GPI-80 antibodies may minimally enrich HSCs further, which is hard to assess quantitatively given limited available data. Namely, CD41 was absent in the VE-CAD^+^CD45^+^ population of a CS14 embryo and therefore would not contribute to enrichment, whereas in a CS16 embryo, CD41^+^ cells constitute approximately 4%, which would potentially give minimal enrichment.

Further analysis showed a tiny cell fraction co-expressing GPI-80 and c-KIT in the HSPC1 population after CS13, at the appearance of the first definitive HSCs. Although midsemester foetal liver-derived HSCs express GPI-80 (Prashad et al., 2015; Vanuytsel et al., 2022), only GPI-80-negative fractions from the AGM region and early embryonic liver were capable of long-term repopulation. Due to low frequency of GPI-80^+^ cells (2-7%) within the VE-CAD^+^CD45^+^ population, negative enrichment with this marker can only be minimal (Fig. 2A). Our data do not allow us to fully exclude that at CS18 liver HSCs may express *VNN2* transcripts in the absence of the GPI-80 protein (Vanuytsel et al., 2022). At which stage during development liver HSCs become GPI-80 positive and whether this is associated with changes in HSC biology requires further investigation.

Long-term multi-lineage repopulation analysis demonstrated that HSCs both from the AGM region and the liver are capable of generating innate lymphoid and dendritic cells. Whether HSCs contribute into the foetal pool of innate lymphoid cells and dendritic cells or these cells are mainly generated by the yolk sac-derived innate lymphoid biased progenitors (Ni et al., 2024) remains an open question. During foetal life, HSCs contribute minimally at least into erythropoiesis (Soares-da-Silva et al., 2021).

To date, the timing of liver colonisation by HSCs in the human embryo has not been clearly determined. A dramatic tenfold increase of *in vitro* clonogenic progenitors (CFU-Cs) in the liver occurs during CS14 to CS15 suggesting its early colonisation (Easterbrook et al., 2019). However, in our previous studies, none of 21 livers from CS14-16 embryos gave long-term engraftment of NSG recipients, and only one out of seven livers at CS17 resulted in long-term multi-lineage haematopoietic engraftment (Ivanovs et al., 2011, 2020). Additionally, we observed poorly interpretable repopulation by two cultured livers (CS17 and CS16), in the latter case with minimal bone marrow chimerism lacking peripheral blood contribution (Easterbrook et al., 2019). In the current study, we report about one more case of a strong long-term multi-lineage repopulation by CS18 liver cells, cumulatively providing firm evidence that CS17-18 is the critical stage of liver colonisation by HSCs (with possible variations depending on genetic background). This is the stage when IAHCs disappear from the dorsal aorta, likely marking HSPC relocation to the liver (Tavian et al., 1999). In agreement with our finding, cells with the HSC signature were detectable in CS17, but not CS15, liver (Calvanese et al., 2022).

In addition to multi-lineage repopulation, we observed uni-lineage long-term T-cell repopulation by CS18 liver cells, similar to our previous report of uni-lineage repopulation by two CS17 livers (Ivanovs et al., 2011). To our knowledge, such observations have not been reported in other vertebrate species, although transient HSC-independent T-cell waves have been described in zebrafish and mouse embryos (Luc et al., 2012; Tian et al., 2017). The origin of long-term repopulating T-cells emerging in the human liver prior to thymus colonisation and their place in the developing haematopoietic hierarchy remain to be determined.

In summary, we show that the first definitive HSCs emerging in the human AGM region, in addition to previously established markers, also express CD43^+^ but not CD41 or GPI-80. The inclusion of these markers enables approximately 1:200 enrichment of HSCs within the basic VE-CAD^+^CD45^+^ population, bringing more certainty in the phenotype of the first definitive HSCs. Our current data together with previous studies (Ivanovs et al., 2011, 2020) firmly establish that HSC colonisation of the embryonic liver occurs during CS17/18. Further deeper analysis of the first HSCs will improve our understanding of the mechanisms driving their development and potentially facilitate HSC generation from pluripotent stem cells for therapeutic applications.

### Study limitations

How confident can we be that the markers tested in transplantations reflect actual phenotype of the first HSCs, given limited number of transplantations due to limited availability of human embryonic material? There is probably a certain specific phenotype for definitive HSCs in the AGM region. If so, our analysis determines this phenotype because each of three markers of interest was tested in two or three independent transplantation experiments and showed consistent results. Even if phenotypically different HSCs are found in the AGM in future, this would not invalidate but rather expand our current conclusions.

Our transcriptomics analysis revealed specific markers expressed in the VE-CAD^+^CD43^+^CD41^−^ population enriched for HSCs. These include the arterial marker GJA5, suggesting the closeness of these cells to the arterial endothelium, and the homeobox transcription factor NKX2-3. Are these markers expressed in HSCs? This remains unclear due to the rarity of HSCs in the VE-CAD^+^CD43^+^CD41^−^ population. While in future, the expression of cell-surface GJA5 in AGM-derived HSCs may be assessed by transplantation of sorted cells, testing NKX2-3 expression in HSCs will be more challenging.

## MATERIALS AND METHODS

### Human embryonic tissues

Human embryos were obtained immediately after medical elective termination of pregnancy. Informed written consent to use the samples in research was obtained from patients. Specimens were provided by the Centre for Reproductive Health (study approved by the Lothian Research Ethics Committee) and by the Joint MRC/Wellcome (MR/R006237/1) Human Developmental Biology Resource (https://www.hdbr.org/). Human embryos were staged using anatomic criteria and the Carnegie classification (O'Rahilly and Müller, 1987; Tavian and Peault, 2005). Samples were either used immediately for fluorescence-activated cell sorting (FACS) analysis and the long-term repopulating assay, as previously described (Ivanovs et al., 2011), or fixed in PBS supplemented with 4% paraformaldehyde (Sigma-Aldrich), embedded in gelatine and stored at −80°C (for immunofluorescence).

### Animals and long-term repopulation assay

NSG mice were bred within the University of Edinburgh, Edinburgh, UK. Experiments with mice were approved by the Animal Welfare committee of the University of Edinburgh, Scotland, UK. Mice were kept under specific pathogen-free conditions in individually ventilated cages. Up to 6 h before transplantation with human cells, 6- to 8-week-old female NSG mice received a sub-lethal total body irradiation dose of 3.5 Gy at a rate of 0.75 Gy/min from a ^137^Cs source (GSR D1 γ-irradiator, Gamma-Service Medical). Animals were transplanted with cells intravenously via the tail vein.

### Flow cytometry analysis

The following mouse anti-human monoclonal antibodies were used for flow cytometry analysis: CD3-APC, PE and PerCP (clones SK7 and SP34-2, BD Biosciences), CD4-APC and APC-Cy7 (clone RPA-T4, BD Biosciences), CD8-PE and PE-Cy7 (clone RPA-T8, BD Biosciences), CD11b-PE-Cy7 (clone ICRF44, BD Biosciences), CD13-APC (clone WM15, BD Biosciences), CD14-APC and APC-Cy7 (clones M5E2 and MϕP9, BD Biosciences), CD19-PE (clone HIB19, BD Biosciences), CD33-PE (clone WM53, BD Biosciences), CD34-APC (clone 8G12, BD Biosciences), CD41a-FITC and PE (clone HIP8, BD Biosciences), CD43-PE (clone 1G10, BD Biosciences), CD45-Biotin, FITC and V450 (clone HI30, BD Biosciences), CD66b-FITC (clone G10F5, BD Biosciences), CD94-APC (clone HP-3D9, BD Biosciences) and CD235a-APC (clone GA-R2, BD Biosciences), VE-cadherin-PE (clone TEA 1/31, Beckman Coulter), C-Kit-APC (clone A3C6E2, Miltenyi Biotec) and GPI-80-PE (clone 3H9, MBL International). Human FcR Blocking Reagent (Myltenyi Biotec) and anti-mouse CD16/32 purified monoclonal antibody (clone 93) (eBioscience) were used to prevent unwanted binding of antibodies to Fc receptors. All antibodies and reagents listed above were used at final concentrations either recommended by manufacturers or determined by titration in-house. 7-amino-actinomycin (7-AAD) (eBioscience) was used for dead cell exclusion. FACSCalibur and LSRFortessa (both from BD Biosciences) instruments were used for flow cytometry analysis. Flow cytometry data were analysed with FlowJo v.7.6.1 software (Tree Star).

### Magnetic cell separation

In our previously published human AGM-derived HSCs transplantations, we employed magnetic cell separation (Ivanovs et al., 2011, 2014) as our initial transplantations using FACS-purified cells had failed to engraft recipient animals. In the present study, cell populations of interest were purified using MACS technology (Miltenyi Biotec). CD43^+^, CD41^+^ and GPI-80^+^ cells were isolated using anti-PE MicroBeads. Prior to this, cells were labelled with mouse anti-human CD43-PE (clone 1G10, BD Biosciences), CD41a-PE (clone HIP8, BD Biosciences) or GPI-80-PE (clone 3H9, MBL International) monoclonal antibodies. Magnetic cell separation was performed manually using a QuadroMACS separator and LS columns. Cell labelling, washes and isolation were performed in accordance with the manufacturer's recommendations.

### Immunofluorescence

The techniques used have been previously described (Sinka et al., 2012). Briefly, 5-μm-thick sections were incubated with primary antibodies overnight at 4°C, then for 1 h at room temperature with biotinylated secondary antibodies and, finally, with fluorochrome-labelled streptavidin. Tyramide Signal Amplification (TSA) Fluorescence Plus System (Akoya Biosciences) was used to reveal CD43 expression. The anti-human uncoupled primary antibodies were: anti-human CD34 (QBEnd/10, Sigma-Aldrich), anti-human CD43 (DF-T1, SouthernBiotech). Secondary biotinylated antibodies were: goat anti-mouse IgG (Jackson ImmunoResearch). For double-immunofluorescence staining, we used Dylight488-coupled streptavidin (BioLegend) and the TSA Fluorescent Plus System. An isotype-matched negative control was performed for each immunostaining. The stained sections were coverslipped in Prolong Gold Antifade Mountant with DAPI (Thermo Fisher Scientific) and analysed with an Axio Imager M2 microscope (Zeiss) coupled to an ORCA-Flash4.0 V3 camera (Hamamatsu Photonics) using the ApoTome.2 function for optical sectioning.

### Whole-mount embryo immunostaining

Whole-mount immunostaining with benzyl alcohol/benzyl benzoate (BABB) was performed as previously described (Yokomizo et al., 2012) with modifications as described below. Human embryos were fixed in 2% PFA/DPBS for 1 h at 4°C followed by dehydration in increasing concentrations of methanol (50%, 75%, 100%). Then, the AGM region was dissected and rehydrated in 50% methanol/DPBS, washed with DPBS and blocked in DPBS/0.2% Triton X-100/50% serum (human and mouse). The tissue was then stained overnight with primary antibodies diluted with DPBS/0.4% bovine serum albumin/0.4% Triton X-100 on a rocker at 4°C. Primary antibodies used were mouse anti-human CD144-PE (TEA 1/31, Beckman Coulter), unconjugated rabbit anti-human CD41 (EPR4330, Abcam) and rat anti-human CD45 (YTH24.5, Abcam) followed by overnight staining with the secondary antibodies: goat anti-mouse Alexa Fluor 546, donkey anti-rabbit Alexa Fluor 488, goat anti-rat Alexa Fluor 647 (all Invitrogen). After a series of washes, the AGM was dehydrated with 50% and then 100% methanol/DPBS and cleared with BABB solution. Images were acquired with an inverted confocal microscope (Leica SP8) and processed using Volocity and Fiji software.

### Bioinformatic analysis

Raw sequencing data from selected published datasets (Table S1) were downloaded from Gene Expression Omnibus (accession numbers GSE135202, GSE162950, GSE233132 and GSE151876). These were aligned against the human reference genome GRCh38-3.0.0 and quantified using the CellRanger Single cell software suite (v.3.1.0, 10x Genomics). The resultant gene expression count files were processed using the Seurat pipeline and quality control for each dataset performed using the same metrics utilised in the original paper. Following quality control, all the datasets were integrated, following Seurat's Integration vignette. This was followed by dimension reduction and unsupervised clustering. Using the DEGs, we grouped the clusters into five broad cell types. The HSPC population was further subsetted to investigate the different subtypes present.

## Supplementary Material

10.1242/develop.205108_sup1 Supplementary information

Table S4. Differentially Expressed Genes between clusters HSPC1 and HSPC2. Surface/secreted factors and transcription factors are indicated in columns I and J, respectively

Table S5. Differentially Expressed Genes between clusters VE-CAD+CD41+ (2-1635); VE-CAD+CD43+ (1636-3865) and VE-CAD+GPI-80+(3866-5358) Surface/secreted factors and transcription factors are indicated in columns I and J, respectively
