# Supplementary material for: CD43, but not CD41 or GPI-80, marks first definitive haematopoietic stem cells in the human embryo
Source: Development. 2025 Nov 20;152(22):dev205108. doi: 10.1242/dev.205108 (PMC12669970; doi:10.1242/dev.205108)
Supplement: Supplementary information [file develop-152-205108-s1.pdf]

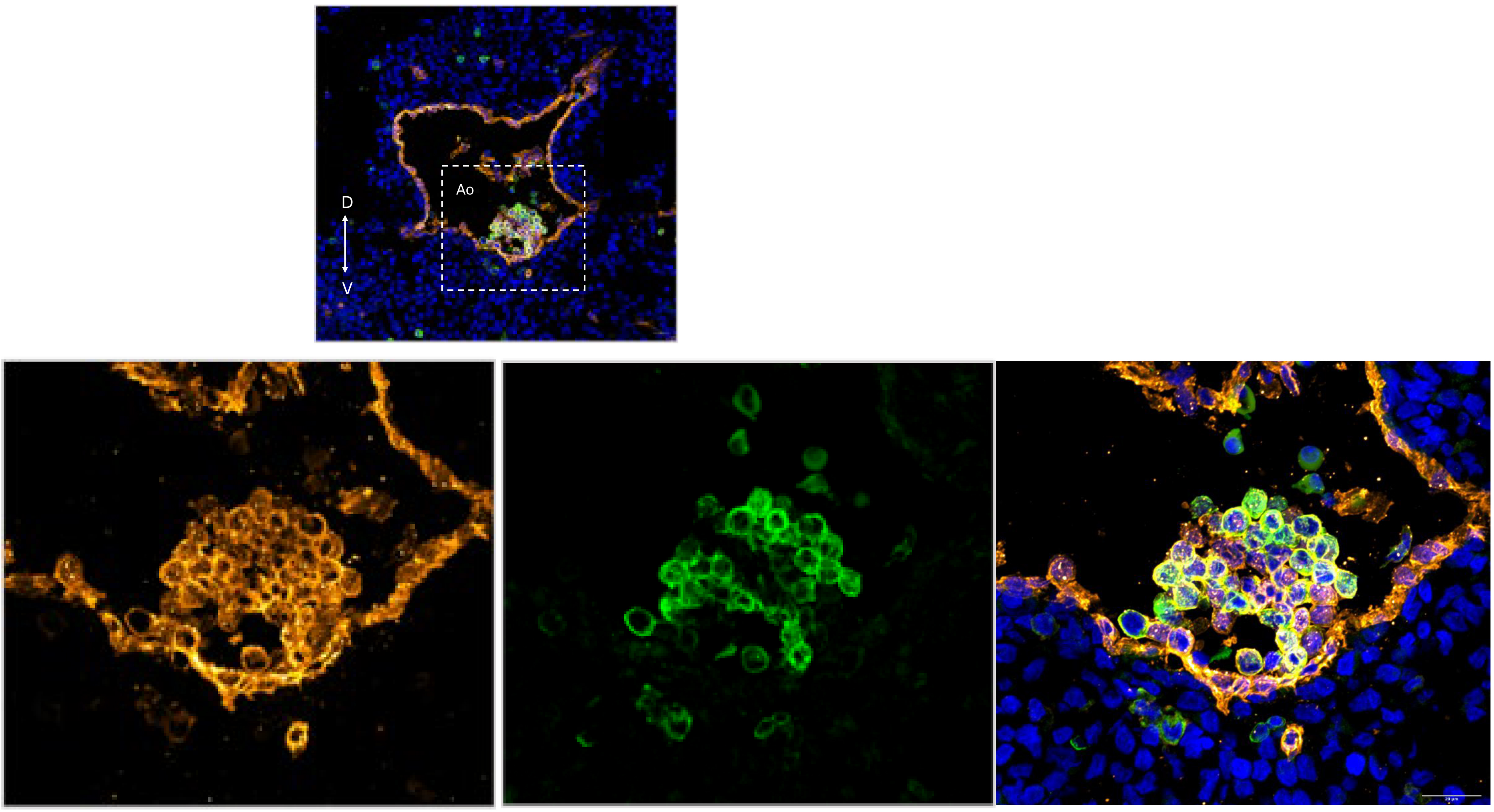

Fig. S1. CS15 human embryo transverse sections showing intra-aortic cluster inside aorta (Ao) lumen.
Stained with sheep anti-human CD31 (orange; R&D Systems, polyclonal; secondary antibody anti-sheep Alexa Fluor 568), mouse anti-human CD43 (green; Thermofisher, clone DF-T1; anti-mouse Alexa Fluor A488) and DAPI.

Development • Supplementary information

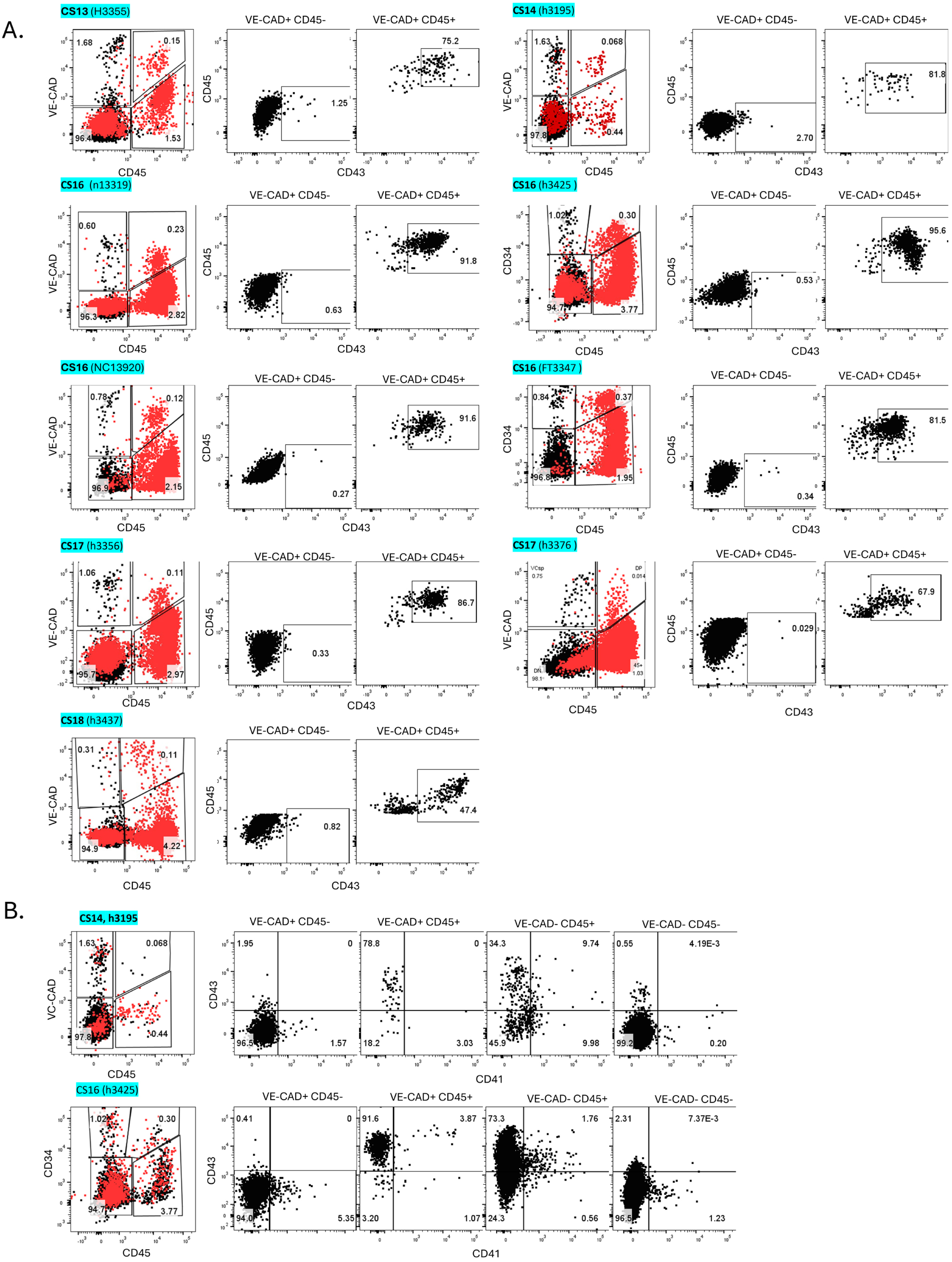

Fig. S2. Distribution of CD43+ and CD41+ cells at different Carnegie Stages in individual embryos
A. CD43+ cells (red or specifically gated).
B. CD41+ cells (red or specifically gated).
Carnegie Stages are highlighted by blue. For additional details for AGM/ dorsal aorta dissections and total cell numbers see Table S1.

Development • Supplementary information

Table S1. (related to Fig. 1 A,B) Cell populations across different Carnegie Stages

Carnegy Stage	Tissue	Total cells	VC+CD43+CD45-	VC+CD45+	VC+CD43+CD45+	CD43+	CD41+	GPI-80+
		cells/ tissue						
CS13	AGM	100501	24	127	96	563	nd	2556
CS14	AGM	106400	30	66	56	918	312	nd
CS15	Ao	255760	nd	278	nd	nd	nd	nd
CS16	AGM	290467	18	672	617	20104	nd	2145
CS16	AGM	258000	13	764	721	16902	2269	nd
CS16	Ao	310000	6	783	638	4613	nd	nd
CS16*	Ao	335000	5	286	271	4471	nd	nd
CS17	AGM	360000	11	366	320	18288	nd	4045
CS17	CP	2345146	3	297	201	16158	nd	2363
CS18	Ao	482330	8	416	197	17576	nd	3022

AGM - aorta-gonad-mesonephros region

Ao - dorsal aorta

CP- caudal part

* - Ao from embryo NC13920 was dissected in the dorsal and ventral parts, which might cause some loss of cells from intra-aortic clusters

Total numbers of CD43+, CD41+, and GPI-80+ cells per each sample (independently of presence or absence of other markers) are indicated in 3 left columns, respectively

Table S2. Datasets employed for single-cell transcriptomics analysis in current study

Public Dataset	Tissue	Stage	SequencingPlatform	Lane_ID	AccessionCode	Paper
Human embryonic AGM	Body	CS10	10X	GSM3993420	GSE135202	Zeng et al Nature 2019
Human embryonic AGM	Caudal Half	CS11	10X	GSM3993421	GSE135202	Zeng et al Nature 2019
Human embryonic AGM	Dorsal Aorta	CS13	10x	GSM3993422	GSE135202	Zeng et al Nature 2019
Human embryonic AGM	AGM	CS14	10X	GSM4968831	GSE162950	Calvanese et al Nature 2022
Human embryonic AGM	AGM	CS15	10X	GSM4968832	GSE162950	Calvanese et al Nature 2022
Human embryonic AGM	AGM	CS16	10X	GSM4968833	GSE162950	Calvanese et al Nature 2022
Human embryonic AGM	AGM	CS17	10X	GSM4968834	GSE162950	Calvanese et al Nature 2022
Human embryonic AGM	Dorsal Aorta	CS13	10x	GSM7401883	GSE233132	Crosse et al, Development 2023
Human embryonic AGM	AGM	CS16	10X	GSM4592621	GSE151876	Crosse et al, Cell Stem Cell 2020

CS=Carnegie stages

All datasets can be accessed from GEO using the Lane_ID and Accession code

Table S3. Marker genes utilized in cluster annotation

Cluster	Marker
HSPC	RUNX1, CD34,SPINK2,HLF,MECOM,HOXA9,MLLT3
Hem	SPN,CD34
Endo	CDH5
Epi	EPCAM
Stroma	COL1A1,PDGFRA

Table S4. Differentially Expressed Genes between clusters HSPC1 and HSPC2.

Surface/ secreted factors and transcription factors are indicated in columns I and J, respectively

Available for download at
https://journals.biologists.com/dev/article-lookup/doi/10.1242/dev.205108#supplementary-data

Table S5. Differentially Expressed Genes between clusters VE-CAD+CD41+ (2-1635); VE-CAD+CD43+ (1636-3865) and VE-CAD+GPI-80+ (3866-5358) Surface/ secreted factors and transcription factors are indicated in columns I and J, respectively

Available for download at
https://journals.biologists.com/dev/article-lookup/doi/10.1242/dev.205108#supplementary-data

Development • Supplementary information
